# Continuous microfluidic flow-through protocol for selective and image-activated electroporation of single cells†

**DOI:** 10.1039/d3ra03100d

**Published:** 2023-06-27

**Authors:** Felix Pfisterer, Neus Godino, Tobias Gerling, Michael Kirschbaum

**Affiliations:** a Fraunhofer Institute for Cell Therapy and Immunology IZI, Branch Bioanalytics and Bioprocesses IZI-BB Am Muehlenberg 13 14476 Potsdam Germany michael.kirschbaum@izi-bb.fraunhofer.de

## Abstract

Electroporation of cells is a widely-used tool to transport molecules such as proteins or nucleic acids into cells or to extract cellular material. However, bulk methods for electroporation do not offer the possibility to selectively porate subpopulations or single cells in heterogeneous cell samples. To achieve this, either presorting or complex single-cell technologies are required currently. In this work, we present a microfluidic flow protocol for selective electroporation of predefined target cells identified in real-time by high-quality microscopic image analysis of fluorescence and transmitted light. While traveling through the microchannel, the cells are focused by dielectrophoretic forces into the microscopic detection area, where they are classified based on image analysis techniques. Finally, the cells are forwarded to a poration electrode and only the target cells are pulsed. By processing a heterogenically stained cell sample, we were able to selectively porate only target cells (green-fluorescent) while non-target cells (blue-fluorescent) remained unaffected. We achieved highly selective poration with >90% specificity at average poration rates of >50% and throughputs of up to 7200 cells per hour.

## Abstract 2

Electroporation of cells is a widely-used tool to transport molecules such as proteins or nucleic acids into cells or to extract cellular material.

## Graphical abstract

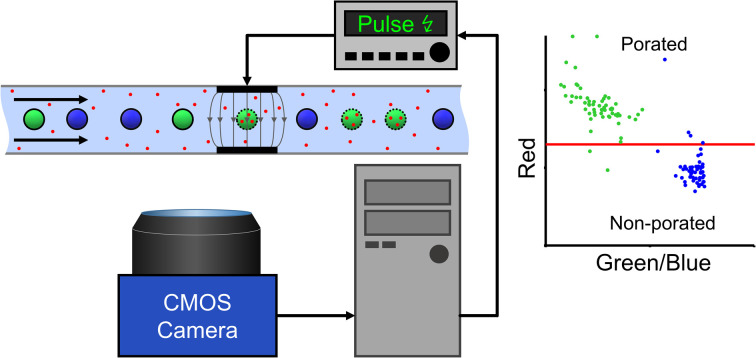

## Introduction

Electroporation is a widely used tool to transport molecules such as nucleic acids or proteins across the cell membrane, to extract cell components or to eliminate unwanted cells from heterogeneous cell samples.^1–4^ It is used, for example, in cancer therapy to modify specific subtypes of T cells, to generate tumor antigen-presenting dendritic cells or to destroy malignant cells.^5,6^ Electroporation-based transfection is also widely used in industry or basic research, either for biomolecule production or for generating transgenic mice.^4,7^ Moreover, poration-mediated extraction of cell contents,^8^ in combination with omics technologies, can provide insight into any metabolic process in the cell.^9,10^ The starting point for most of these applications is heterogeneous cell samples, where only the selected target cells should be treated, while the other cells must remain unaffected.

Standard bulk electroporation offers high efficiency, robustness and flexibility in adapting to different cell types, buffers and targets. Alternatively, microfluidic approaches have been developed that allow membrane permeabilization on the level of the individual cell. This is achieved by driving the cells through a narrow channel that increases shear force and causes mechanoporation, or using integrated electrodes for electroporation.^11–13^

However, none of the describe methods provides the ability to permeabilize individually selected target cells from heterogeneous cell samples without presorting (*e.g.*, using FACS). The latter is often associated with cell damage, cell loss, high investment costs, or is simply not applicable, as is often the case in the therapeutic context.^14^ Alternatives such as micro- and nanoscale technologies offer single-cell precise poration by employing nanopillars, optical tweezers or nano-straws. However, they are very labor-intensive and tedious and do not provide the throughput needed for industrial or medical applications.^15–19^

To overcome these limitations, we present a microfluidic approach for the selective poration of predefined target cells in heterogeneous cell samples based on microscopic observation and real-time image analysis. We employ a commercial camera system to inspect cells flowing along a microfluidic channel. The cell's characteristics are automatically analyzed to decide whether or not to porate the cell between a pair of electrodes placed downstream.

As a proof of concept, we selectively electroporated cells labeled with a specific fluorescence color in a sample of differently stained cells, while leaving the non-target cells unaffected. Independent on the applied pulse voltage, we achieved highly selective cell poration of the target cells with specificities around 90% at throughputs of 7200 cells per hour and sensitivities of at least 50%. Live/dead staining showed that cells were vital 3 days after treatment. The low complexity and ease of use make our microfluidic dieletrophoretic poration approach unique since it only requires an ordinary microscope, a fluidic system and a pulse- and signal generator for operation which could be parallelized due to the small footprint of the electroporation unit. Finally, it offers a high degree of flexibility regarding the targeted cell type due to its image-based control, and it even allows individual pulse protocols for different target cell types in the same sample.

## Results

### Microfluidic chip and microfluidic protocol

**Fig. 1:**
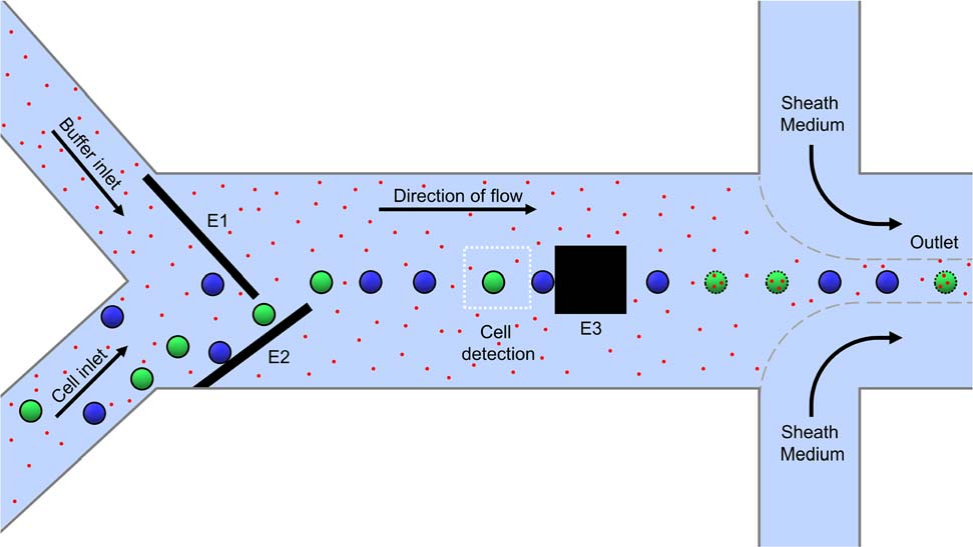
Schematic representation of the microfluidic channel (2D top view). Cells and electroporation buffer enter the channel in parallel *via* separate inlets. Top and bottom slides of the microchannel are patterned with congruent DEP microelectrodes (E_1_, E_2_) operated in negative (*i.e.*, cell-repelling) DEP mode, forcing the cells into a single line. As a result, they sequentially enter the cell detection area, where they are analyzed and classified by color (target and non-target cells are green and blue fluorescent, respectively). The cells are then hydrodynamically forwarded to the poration electrode (E_3_), where an electric pulse is applied only in the case of a target cell. Porated target cells are indicated with a dotted perimeter and cargo with red dots. After that, the cells are flushed out of the chip with the help of a sheath flow and re-collected in standard microplates.

A 2D schematic of the top view of the microfluidic channel for electroporation is shown in Fig. 1. It has a height and width of 35 μm and 650 μm, respectively and it is made by sandwiching the channel structure made out of photoresist between two glass substrates. The glass substrates are patterned with microelectrodes for dielectrophoretic cell handling (see Methods section). The microfluidic system has one sample inlet for cells suspended in poration buffer and one additional buffer inlet to allow the online adjustment of the cell density in the main channel. Besides this, there is a sample outlet flanked by two additional inlets of sheath flow. The latter are used for a quick and efficient sampling out of the cells, as the total flow increases from 30 μL h^−1^ to 1000 μL h^−1^.

**Fig. 2:**
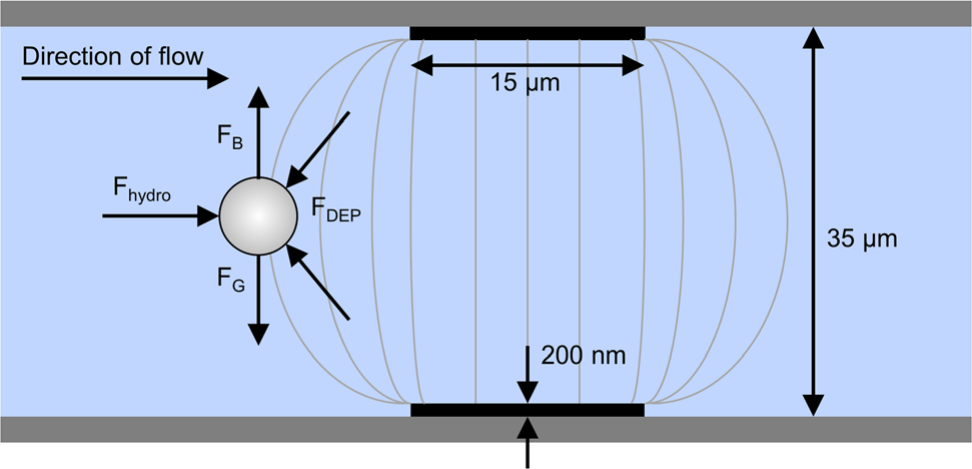
Side view of the microchannel with electrodes on top and bottom and schematic representation of electric field lines (not to scale). The DEP force contains components acting in both horizontal and vertical direction, which counteracts not only the hydrodynamic force (*F*_hydro_) but also gravitational (*F*_G_) buoyancy force (*F*_B_). Thus, a horizontally deflected cell is focused towards the channel center.

Cells enter the chip through the cell inlet and are lined up by two deflection electrodes (Fig. 1, E_1_ and E_2_). Due to the special electrode configuration in our system (see Fig. 2), the repulsive dielectrophoretic force contains a component both acting in the horizontal and vertical direction, counteracting not only the hydrodynamic force (*F*_hydro_), but also gravitational (*F*_G_) and buoyancy force (*F*_B_). Thus, sedimented cells are lifted from the channel bottom towards the channel center^20^ while being deflected (Fig. 2). This ensures that the cells leave the deflection electrodes and enter the cell detection area centered in the focal plane of the microscope.

Image acquisition takes place in a conventional fluorescence microscope, where both fluorescence signals or transmission images can be used for cell classification (enabling for example cell classification based on fluorescent protein expression levels, fluorescently-labeled surface markers, or morphological parameters like cell size and shape). In particular, for the present experiments and as a proof of concept, the cells are detected and classified by cytosolic fluorescence staining as a model for fluorescent protein expression.

Only the cells classified as target (depicted green in Fig. 1) were pulsed between two pulse electrodes (E_3_) downstream of the detection area for electroporation, while the non-target cells (blue) remained untreated. Due to their small size of only 50 μm × 50 μm, the poration electrodes allow selective and individual poration when cell spacing is 50 μm at minimum. This results in a theoretical maximum processing capacity of approximately five cells per second at 370 μm s^−1^ (equals 18,000 cells per hour at 30 μL h^−1^). Finally, after leaving the poration area, the cells are driven to the outlet and flushed from the chip in a controlled manner using a sheath flow to be collected in a 96-well plate.

### Synchronization of cell movement and pulse application with optical LED feedback

**Fig. 3:**
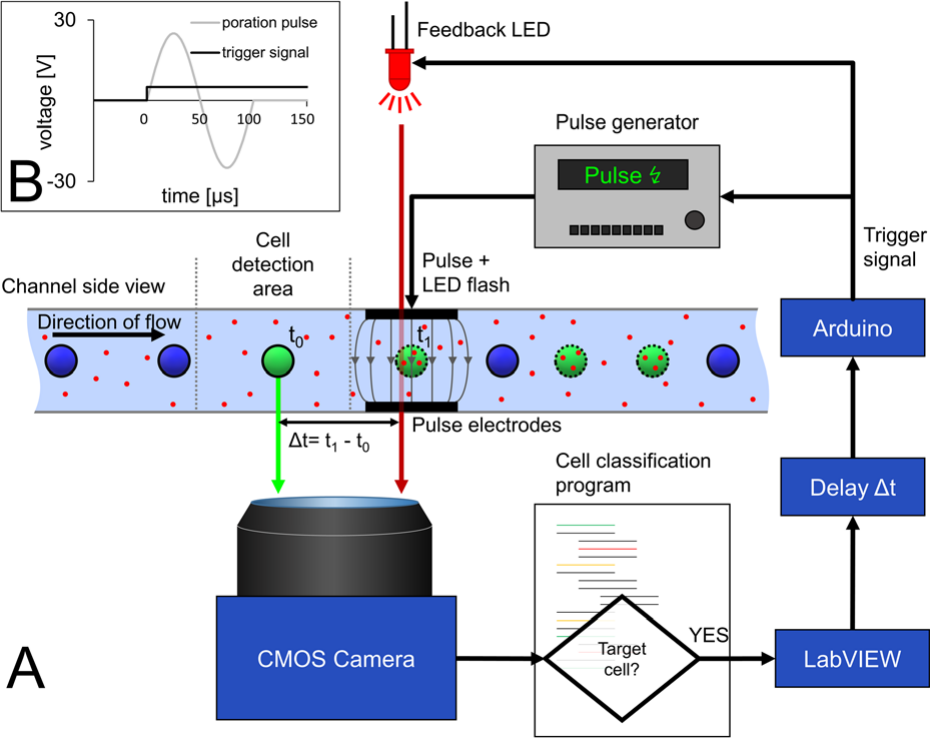
Schematic of the set-up for triggered pulse application and optical feedback mechanism for determining pulse delay. (A) When a green-stained cell enters the cell detection area, a custom Python script that constantly analyzes the camera images identifies the cell as target and activates a LabVIEW script. The latter sends a command to an Arduino board, which in turn sends a trigger signal to a function generator to generate a sine wave pulse for electroporation (see B). Since the cell takes some time to travel from the detection area to the poration electrodes (Δ*t* = *t* − *t*_0_), the LabVIEW script is triggering the pulse signal with a delay, which is dependent not only on velocity of the cell but also on the latency in image acquisition, image processing and communication between hardware and software modules. For quantification of this delay, we developed an optical feedback mechanism that provides optical feedback on the exact time of occurrence of the pulse. For that, the Arduino board not only triggers the function generator but also drives an LED in parallel, which feeds a light flash into the optical path at the time point of electric pulse application. The light flash appears in the camera image and can thus be precisely matched to the position of the cell in the microchannel (see A). Porated target cells are indicated with a dotted perimeter and cargo with red dots.

The decision to pulse a cell is made after the analysis in the cell detection area directly in front of the pulse electrode (Fig. 1, E_3_). Images are acquired at a frame rate of 100 fps, which corresponds to a camera cycle of 10 ms. As schematized in Fig. 3, a custom-made Python script reads the frame, detects and tracks the cells, excluding cell clusters, and analyzes the color for each camera cycle. When a target cell is detected at a certain distance from the poration electrode, a message is sent to the main Labview controller software *via* an internal TCP/IP communication. Afterwards, an Arduino also controlled by LabVIEW generates a trigger signal for the pulse generator with a time delay (Δ*t*) to allow the cell to get exactly between the pulse electrodes before pulse application. The pulse generator generates a predefined sinusoidal pulse that is fed into the pulse electrodes.

Since the cell is in motion and is only between the pulse electrodes for the short time period of 80 ms, precise determination of pulse delay after cell detection is a key challenge. Not only the time required for the cell to move from the detection area to the pulse electrodes must be considered but also the latency in image acquisition, image processing and communication between the devices must be compensated for. This makes it hard to estimate the correct time delay theoretically.

To ensure the synchronization between the poration pulse and the presence of the target cell between the pulse electrodes, an optical feedback system was established. For that, the same signal that triggers the pulse generator was used in parallel to drive an LED that illuminates the region of interest to provide accurate optical feedback on when the pulse occurs (Fig. 3). In this way, the necessary pulse delay can be experimentally adjusted. The position of the cell at the time of LED pulse application can then be observed in the video stream to verify that the pulse delay is applied exactly when the cell is between the pulse electrodes (Fig. 3A and video in ESI†). This delay is dependent on the flow rate and calculates to 75 ms for 370 μm s^−1^. The determination of the delay needs to be done once and kept constant for subsequent experiments with the same flow rate.

**Fig. 4:**
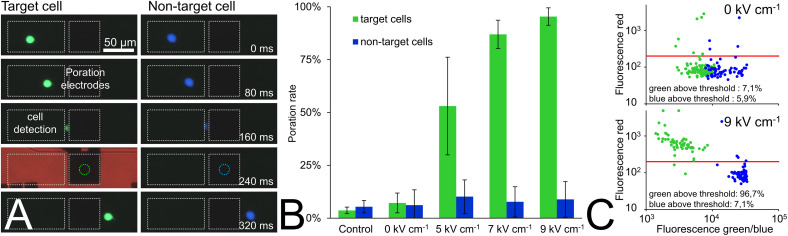
Synchronization of pulse application and results of color-based cell poration. (A) Image sequence of a green fluorescent (target) cell and a blue fluorescent (non-target) cell flowing through the microchannel at 370 μm s^−1^ (Δ*t* between individual frames, 80 ms). The movement of the cells is clearly visible until they disappear between one of the poration electrodes (here, their anticipated position is marked with a dotted circle). In case of the green (target) cell, the red optical feedback flash in the moment of pulse application appears exactly when the cell is anticipated between the poration electrodes. In contrast, the blue (non-target) cell passes the electrodes without being pulsed. (B) A heterogeneous mixture of green and blue fluorescent cells were flushed through the chip. Target cells were either electroporated with pulses of 100 μs length and various electric field strengths (given in RMS) or did not receive any pulses (*i.e.*, 0 kV cm^−1^). PI in the channel medium entered the cell upon membrane poration and, thus, served as poration indicator. After treatment, cells were recollected from the chip in microplates and the amount of PI-positive cells (*i.e.*, poration rate) was determined in the fluorescence microscope. As a control, a sample from the cell culture bottle was diluted comparably to the samples from the chip and analyzed in the same way. (C) Mean red fluorescence intensities of green- and blue-stained cells before (top) and after (bottom) treatment in the chip as described above (exemplary data at 9 kV cm^−1^). The red line indicates the threshold of 200 below which the fluorescence value of 95% of the non-pulsed cells is and above which a cell is counted as porated.

With our system, we selectively electroporated target cells from a heterogeneous cell sample (Fig. 4). For that, we flushed a mixture of blue and green fluorescently stained cells with a ratio of 1 : 1 through the chip. Green stained cells were defined as target cells, blue stained cells as non-target cells. Propidium iodide (PI) was added to the channel medium, serving as an indicator to measure the cell poration depicted as red dots in Fig. 1. This dye is membrane-impermeable in intact cells but can enter the cytoplasm through a porated cell membrane and start to fluoresce in red color upon intercalation in nucleic acids.

There had to be sufficient distance between the lined-up cells to reduce the probability of more than one cell being pulsed at the same time. This was ensured by adjusting the cell concentration such that at the applied flow velocity of 370 μm s^−1^, 0.2 to 2 cells per second passed between the pulse electrodes. This corresponds to a mean cell distance of 185–1850 μm (*i.e.*, more than three times larger than the poration electrode diameter). Single sinusoidal electrical pulses with a period length of 100 μs and field strengths of 5, 7, or 9 kV cm^−1^ root mean square (RMS) were applied for cell poration. A sheath flow of conditioned cell culture medium was used to flush the cells out of the fluidic system and to recollect them in a 96-well plate. The amount of intracellular nucleic acid-bound PI was subsequently quantified semi-quantitatively in both blue and green stained cells with an automated fluorescence microscope (see Methods section). Processed samples were compared with non-pulsed controls (*i.e.*, cells from the cell culture flask (control, Fig. 4B) or cells that were driven through the chip but did not experience any pulse (0 V condition, Fig. 4B)).

At a low field strength of 5 kV cm^−1^ RMS, the poration rate (*i.e.*, amount of PI-positive cells) of the green cells was 53% and increased to 87% and 95% when using 7 kV cm^−1^ and 9 kV cm^−1^ RMS, respectively. In contrast, only a slightly higher amount of porated cells (about 4%) was observed in the blue non-target cells compared to the non-pulsed cells of both colors in the control or 0 V condition.

As Fig. 4C shows, we observed a strong red fluorescence shift above the arbitrary threshold of 200 (see Methods section) in most of the target cells for a field strength of 9 kV cm^−1^ RMS, while most of the non-target cells or non-pulsed cells (0 kV cm^−1^ RMS) did not exhibit significant red fluorescence (see Fig. 4C).

The performance of the system is particularly evident when considering the selectivity of the poration process in terms of sensitivity and specificity over all experiments (Table 1).

**Table 1:** Sensitivity and specificity of the poration of all processed cells

	Sensitivity (%)	Specificity (%)	Porated target cells	Non-porated non-target cells	Porated non-target cells	Non-porated target cells
Control	3.7	94.7	132	391	22	3474
0 kV cm^−1^	6.0	95.5	152	998	47	2374
5 kV cm^−1^	50.6	89.5	1024	744	87	998
7 kV cm^−1^	85.3	92.5	471	271	22	81
9 kV cm^−1^	92.7	89.7	358	253	29	28

Sensitivity describes the fraction of porated target cells (*i.e.*, green fluorescent, no PI-stain) among all target cells, while specificity describes the fraction of non-porated non-target cells (*i.e.*, blue fluorescent, no PI-stain) among all non-target cells.

The sensitivity increases with increasing pulse intensity. Compared to the background control with just 6% of porated target cells, the sensitivity increases up to 50.6%, 85.3%, and 92.7% for 5, 7 and 9 kV cm 1 RMS, respectively. This indicates that the pulse is applied in all cases but is more likely to lead to poration the stronger it is. The specificity at 0, 5, 7, and 9 kV cm^−1^ RMS is 95.5%, 89.5%, 92.5% and 89.7%, respectively. Thus, the system remains highly selective for the target cells across all pulse strengths tested as specificity keeps between 90 and 95%. This proves that the pulse is delivered very limited in the area of the pulse electrodes and does not affect other cells.

### Vitality rate

**Fig. 5:**
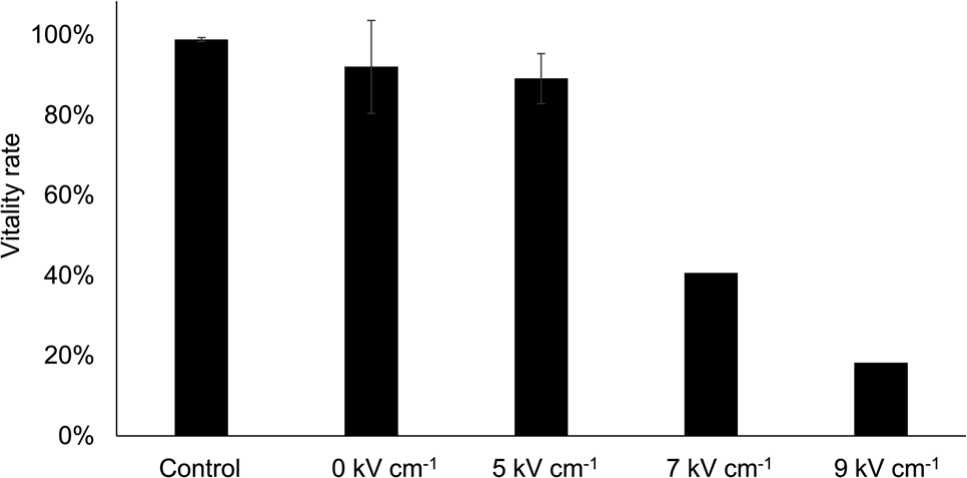
Cell compatibility of the microfluidic electroporation protocol. Unstained cells were introduced into the microfluidic system and electroporated with pulses of various field strengths. We used the same protocol as described in color-based cell poration, but in contrast we used bright-field microscopy for cell detection and porated all the cells in the buffer without addition of PI. Afterwards, the cells were re-collected from the microfluidic system and further grown in cell culture vessels. The amount of vital cells (*i.e.*, vitality rate) was analyzed after 3 or 4 days by staining the cells with calcein green and PI. As a control, we diluted a sample from the cell culture bottle comparably to the samples from the chip and analyzed it in the same way. Field strengths are given in RMS. Experiments were replicated four times (*n* = 4), except for 7 and 9 kV cm^−1^ conditions, which were performed one time each (*n* = 1).

It is well-known, that depending on the intensity of electroporation, some of the cells will not survive the process.^3^ Therefore, we studied the vitality rate of the cells after electroporation (Fig. 5). For this purpose, all cells of a population were pulsed with different field strengths, collected in a 96-well plate and cultured in conditioned cell culture medium for 3 or 4 days. The influence of flushing in and out, *e.g.* by shear forces and by the mere deflection of the DEP electrodes, is reflected by the condition without pulse (0 kV cm^−1^). As a control, a sample directly from the culture flask was diluted to a similar cell density (*ca.* 2000 mL^−1^) and cultivated. To avoid dye-induced effects on vitality rate, unstained cells were used and pulse application was triggered upon cell detection in the bright field image. The vitality rate of the cells was determined by staining them with a live- and dead staining assay of CellTrace calcein green AM and PI and determining the ratio of calcein-positive cells (live) and PI-positive cells (dead).

Flushing and dielectrophoretically deflecting the cells in the channel had only minor effects on the vitality rate. Compared to the control group the vitality rate was only slightly reduced (92% *vs.* 99%, respectively). The same was true for cells that were electroporated with a field strength of 5 kV cm^−1^ RMS, showing a vitality rate of 89%. Those cells pulsed with 7 kV cm^−1^ RMS and 9 kV cm^−1^ RMS, however, showed strongly reduced vitality rates of only 40% and 18%, respectively.

## Discussion

In this work, we present for the first time a microfluidic flow-through system for visually-triggered selective electroporation of single target cells in heterogeneous cell samples. Cells were detected in both fluorescence and transmitted light images. For pulse application, we used 50 μm small thin film microelectrodes present at the inner surface of the microchannel in order to apply electric fields very locally in only a small region between the electrodes. This allows the electric field effects to be applied with pinpoint accuracy to the cell membrane of the target cells without affecting other cells present in the rest of the microchannel. The high selectivity of our approach is clearly demonstrated by the results regarding color-based cell poration shown in Fig. 4 and Table 1.

Here, cells were either pulsed or not at different field strengths during their passage through the microchannel depending on their fluorescence color (green: target cell; blue: non-target cell) and the permeabilization of the cell membrane was visualized *via* a third fluorescence color (red). While the amount of permeabilized cells within the target cells increased with the applied field strength, the amount of porated non-target cells (and with that the specificity or erroneous poration) remained constant close to background level (which is present in each sample, including untreated cells). Thus, only the selected target cells that were located between the two porating electrodes at the time point of pulse application were successfully permeabilized but not neighboring cells or even cells in other regions of the microchannel, demonstrating the high selectivity of our electroporation protocol.

In general, a sigmoidal relationship can be assumed between the poration rate and the applied field strength (see also ref. 21). While in the plateau areas at very low or very high effect strength only small changes in the poration rate with varying field strength are to be expected, this variability is greatest around the half-maximum value. The scatter bars in Fig. 5 show the variability over different experimental runs performed on different days and with different microfluidic chips. Slight differences in channel height, material properties of the porating electrode or other experimental conditions could have resulted in different effective field strengths at the same applied voltage. This would cause the largest deviations in the half-maximum range (*i.e.* around 5 kV cm^−1^), while less pronounced effect variations can be expected at lower or higher voltage regimes.

An intrinsic benefit of employing highly localized electric fields in the microfluidic environment compared to bulk methods is the high control and low variability of the pulse conditions for each cell of the sample. Local variations in field distribution do not come into play in our system. Instead, each cell is treated in the same way as it passes through the poration unit, allowing for highly reproducible results. However, we observed that the poration performance of our chips decreased with the number of experiments performed (data not shown), which noticeably reduced the number of experiments per chip. It is possible that electrochemical processes on the electrodes are responsible for the observed effects by gradually eroding them.

Moreover, as with other bulk methods, we see a trade-off between poration rate and cell vitality rate.^19,22,23^ Although we observed up to 89% vital cells 3 days after electroporation (see Fig. 5), we did not observe considerable cell proliferation from any condition during cultivation period of 5 days (data not shown). Hence, future optimization of our poration buffer and pulse shapes will be necessary to improve the performance and overcome the limitations of our current approach.

In these proof-of-concept experiments, cytoplasmic dyes were used to demonstrate the basic feasibility of the approach, and also being a model system for fluorescent protein marker expression like GFP or CFP. Of course, other relevant biological questions could be addressed that involve cell classification based on the presence or absence of fluorescently labeled surface markers, subcellular fluorescence distribution, co-localization of proteins or even morphological characteristics like size and shape of unlabeled cells detected in transmitted light mode. So working with fluorescently labeled and non-labeled cells is possible with our microscope-based approach, depending on the desired application and cell classification criteria.

We have already performed some image analysis when segmenting the cells from the image, discarding cell clusters and tracking the exact position of each single cell to activate the poration pulse. Employing automated image analysis for cell analysis offers enormous flexibility with regard to the criteria by which a target cell can be defined without the need of modifying the microfluidic flow cell. As mentioned above, it even opens up the possibility to choose between cells considering spatial cues (*i.e.*, high-content features) such as cell shape, subcellular localization of proteins, nuclear/cytoplasmic ratio, and many more.^24–31^

Thanks to the dielectrophoretic control, the cells are flowing in a well-controlled manner along the microchannel at any flow rate. Therefore, the flow velocity can be adjusted easily to allow the necessary computing time even for complex cell classification algorithms on the expense of throughput. Alternatively, the distance between the cell detection area and the position of the poration electrode could be adjusted when longer computing time between image acquisition and pulse application is needed. This flexibility in computational time, even up to hundred milliseconds, gives us the possibility to combine our microfluidic approach with more advanced and complex deep learning algorithms.^32,33^

When spatial cues are used for identifying target cells, motion blur must be taken into account and minimized, depending on the desired image quality. We have worked here with an illumination time of 4 ms, which corresponds to a movement of 1480 nm at a speed of cells of 370 μm s^−1^. At 20× magnification, this corresponds to a traveled distance of about 30 μm on the camera chip during exposure time. With a pixel size of 6.5 μm, this thus generates only moderate motion blur (*ca.* 5 pixels, while a 12 μm-sized cell spans about 37 pixels), which is sufficient for the application shown. However, depending on the desired spatial cue and throughput, this value can be adjusted very easily by reduction of the flow velocity or shortening exposure times in combination with a stronger light source, a more light-intense objective or the use of brighter fluorescent markers.

The present throughput is well above the performance of other selective single-cell poration methods, where only few cells per minute can be processed^34,35^ and is sufficient for research applications (*e.g.* cloning or single-cell sequencing). However, the technically simple combination of DEP electrodes for cell focusing and individually switchable poration electrodes in one system allows easy parallelization by stringing several poration lines side by side within the channel. This could help to increase the throughput of our system, which is currently at 7200 cells per hour with only one poration unit.

One very important advantage of our system is its technologically simplicity. Apart from the microscope and the microfluidic flow cell, it only requires a normal PC, a simple self-developed signal generator, a commercially available function generator and conventional pressure pumps for its operation, which both makes it easy to implement the system in any lab and paves the way for future device development. Moreover, the chip-based approach even allows for future development as an all-in-one disposable solution, which could be attractive for GMP processes, like adoptive T cell transfer or other therapeutic applications. As the microfluidic chip can be easily operated on any type of microscope, our approach can be combined with a wide range of imaging techniques (*e.g.*, epi-fluorescence, phase contrast, holography, Raman spectroscopy *etc.*^36^) to identify target cells, which makes it an interesting general-purpose tool in biotechnology and biomedical research.

## Conclusions

In this work, we present a microfluidic protocol for the visually triggered electroporation of individually selected target cells in heterogeneous cell samples. Cells flow through a microchannel and are guided into the microscopic imaging area by dielectrophoretic forces. There, the target cells are imaged and identified by automated image analysis and, depending on the result of the classification, are pulsed or not pulsed at a poration electrode. Thanks to the image-guided control, the system can be quickly and easily adapted to a large number of different samples and target cell types. In addition, morphological (high-content) features in the cells can be used in the future to identify the target cells. Due to the precisely defined electric field conditions, it offers a high degree of control and selectivity over the electroporation process and thus generates highly reproducible results.

The system is of low complexity and, in addition to the flow cell, only requires a commercially available microscope, a pulse and signal generator, pumps and a PC for control. All this makes it an interesting tool for biomedical research and bio manufacturing, and, in its future version as a disposable cartridge, might be useful also for medical applications.

## Experimental

### Cell culture

Cells of human T cell line Jurkat (ACC 282, DSMZ, Germany) were cultivated at 37 °C and in 5% CO_2_ atmosphere in RPMI 1640 with phenol red, 25 mM HEPES (Pan Biotech, Germany), 2 mM stable l-glutamine (Pan Biotech, Germany) and supplemented with 10% FCS (Biochrom, Germany). Cells which were already processed in the microfluidic chip were cultivated afterwards in conditioned medium prepared as follows: Jurkat cells were cultivated in cell culture medium as described earlier + 100 U mL^−1^ penicillin/streptomycin (Pan Biotech, Germany) with an initial cell density of 10^5^ cells per mL. After 3 days, the supernatant was sterile filtered and mixed with fresh medium at a ratio of 2 : 1 and a total concentration of 1 mM sodium pyruvate (Pan Biotech, Germany).

### Poration buffer

For dielectrophoretic deflection and poration in the microfluidic chip, we used a self-adapted poration buffer as the channel medium in which the cells were previously washed once (300 g, 2 min). The buffer consisted of 220 mM sorbitol (VWR BDH Chemicals, Germany), 25% PBS (Biowest, Germany) and 0.5% PVA (MW approx. 30 000, cas 9002-89-5, Merck, Germany), which resulted in a conductivity of 0.4 S m^−1^ and osmolarity of 300 mOsmol L^−1^. This formulation emerged from preliminary experiments and allows both electroporation and dielectrophoretic deflection of cells.

### Fluidic setup

The microfluidic chip was fabricated by GeSiM mbH, Germany according to our design. We used pressure driven pumps (LineUp series, Fluigent, Germany) with flow sensors (FlowEZ series, Fluigent, Germany) for cell injection, buffer, and sheath flow. The flow rate inside the main channel was 30 μL h^−1^ (370 μm s^−1^) and sheath flow had a combined flow rate of 1000 μL h^−1^. FEP tubing (OD 1.59 mm ID 0.254 mm, Techlab, Germany), PEEK tubing (OD 0.79 mm ID 0.15 mm, Techlab, Germany) and valves (Diba Omnifit, Germany) were used to connect reservoir, chip and pumps. 1.5 mL tubes were used as cell and buffer reservoirs and 15 mL tubes for the sheath flow medium.

### Dielectrophoretic cell handling and chip design

The principle of dielectrophoresis (DEP) is only briefly outlined here, as the effect has been published in detail before.^37–42^ Shortly, DEP occurs when a dielectric particle is placed in an inhomogeneous electric (AC or DC) field. This particle is thus polarized, the strength of polarization depending on the voltage gradient of the electric field, the field frequency and the electrical conductivity and permittivity of the particle and the surrounding medium. By interaction of these charges with the electric field, a force acts on the particle, either directed to the field maximum (attraction towards electrode, positive DEP) or to the field minimum (repulsion from the electrode, negative DEP), the latter being used in our set-up.

In the 35 μm high microfluidic chip, platinum thin film electrodes (width 15 μm, thickness 200 nm) are arranged in congruent pairs at the inner sides of top and bottom glass. They are driven by a custom-built multichannel electrical signal generator, which produced individually switchable square wave signals for each electrode with a frequency of 300 kHz and amplitudes of 3–4.5 V_pp_ (*i.e.*, 0.9–1.3 kV cm^−1^). The control commands are sent *via* USB and are transferred in *ca.* 2 ms for switching all electrodes. Using poration buffer with a conductivity of 0.4 S m^−1^, Jurkat cells experience negative DEP and are repelled from the electrodes. This makes the electrodes act as barriers to the cells, causing them to be deflected from their streamline when electrodes are arranged at an angle to the flow direction.

### Control software

A LabVIEW (National Instruments, USA) interface was the core control system and accomplished the interaction of all hardware devices as well as the communication with the image recognition software written in Python. The signals to trigger the pulse generator as well as the optical feedback LED as pulse indicator were generated using a self-configured Arduino nano board (Arduino, Italy).

### Sample preparation

To measure the efficiency of selective poration of target cells, we used a heterogeneous sample of green (calcein AM, Invitrogen, USA, 1 μM) and blue (calcein violet AM, Invitrogen, USA, 5 μm) stained cells at a ratio of 1 : 1. Staining was performed with a cell density of 3 × 10^5^ to 10^6^ cells per mL at room temperature for 30 min. PI (Acros Organics), a red fluorescent cell membrane impermeable intercalating nucleic acid dye, was added to the buffer at a concentration of 25 μg mL^−1^ as a poration indicator. When the dye enters the cell through the porous membrane, there is a shift in fluorescence maximum upon binding to nucleic acids, which can be detected as an increasing signal in the red fluorescence channel.

### Image acquisition and processing

An inverted fluorescence microscope (cellR, Olympus) was used to observe and control the poration process in the microfluidic chip. It was equipped with a 20× phase contrast objective (Olympus), a triple band filter set (triple band: DAPI, FITC, TxR AHF F62-001 AHF, Germany) for fluorescence imaging and a green illumination (Brightline 531/40, AHF Germany) for transmission microscopy. A CMOS color camera with a pixel size of 6.5 μm (Edge 5.5, PCO, Germany) acquired the microscope images at an exposure time of 4 ms and a frame rate of 100 fps. A custom-written Python program running on a common workstation (Intel Xeon E-2136@3.3 GHz, 32 GB RAM, NVIDIA Quadro P620) processed the images and classified the cells. Fluorescence images were used for the selective cell poration experiments, while unstained cells were analyzed in transmitted light images for the viability assays. There were three main tasks accomplished by the image acquisition and processing software: (i) frame reading and color adjustment, (ii) frame analysis and (iii) communication with the main core software controlled in LabVIEW. For the first task, we used python libraries like CuPy, OpenCV-Python and pco to read the frame from the camera, transform 16 bits to 8 bits frame, and define the dynamic range of the image and finally debayering. The image analysis consisted of cell segmentation, size exclusion to avoid poration of cell clusters, tracking and selection. For the cell segmentation, we used standard morphological transformation, Gaussian filtering as well as color transformation and color and brightness thresholding from the already mentioned OpenCV-Python package. The detected cells were tracked to know the exact position of each of the cells. We computed the distances using functions from scipy.spatial and NumPy, and kept the data in an ordered dictionary from collections. The selection of a target cell is based on color information, in particular, a cell was defined as a target when the contour of the detected cell had a hue value in HSV color space in the range of 50–70 (representing green in a space from 0 to 180). Finally, if a target cell was detected at a certain distance from the poration electrode, a triggering message was sent to the main control software in python *via* a TCP/IP server. The communication was written using the low-level networking interface python package named socket.

### Electroporation

A single sinusoidal electrical pulse with a period length of 100 μs was used for porating target cells. In order to achieve the used peak field strengths of 5 kV cm^−1^ RMS, 7 kV cm^−1^ RMS and 9 kV cm^−1^ RMS, we applied amplitude voltages of 25 V, 35 V and 45 V at an electrode spacing of 35 μm. The pulses were generated with a function generator (33120A, Hewlett Packard, USA) that were amplified fivefold (7602M, Krohn-Hite Corporation, USA) to achieve the required voltages.

### Post-pulse cell analysis

The recovered cells were measured in an automated inverted fluorescence microscope (Olympus IXplore Live with ScanR, 4× objective, Olympus, Hamamatsu digital camera C11440). Fluorochromes were excited at 395/25 nm for blue, 475/28 nm for green and 575/25 nm for red and the emission was measured *via* multiband filter at 438/29 nm, 555/28 nm and 635/22 nm. First, the blue- and green-stained cells were measured (500 ms and 80 ms) and subsequently the relative intracellular PI amount of the detected cells was determined (red channel, 500 ms). Cells were considered porated, when showing intensity values in the red fluorescence channel higher than a threshold of 200. This value was deliberately set very close to a negative control sample, where a majority of more than 95% of the cells would still be considered unporated, so that even a tiny increase in poration rate could be detected.

The vitality rate of the cells was determined by staining them after 3 days cultivation with a live- and dead staining assay of CellTrace calcein green AM (live staining, 1 μM) and PI (dead staining, 10 μg mL^−1^) for 10 minutes at room temperature and determining the percentage of calcein-positive cells in all cells.

Sensitivity and specificity were calculated with the following formulas:
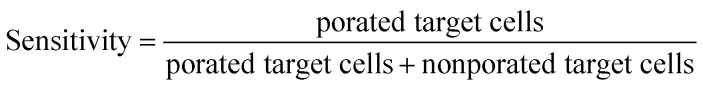

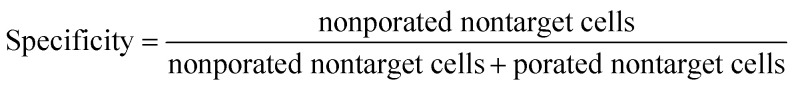


## Author contributions

Concept: FP, MK. Methodology: FP, NG, TG, MK. Software: FP, NG. Validation: FP, NG, MK. Writing and review: FP, NG, MK.

## Conflicts of interest

The authors ensure that there are no conflicts to declare.

## Supplementary Material

RA-013-D3RA03100D-s001
